# A novel isothermal method for amplification of long specific amplicon from linear template

**DOI:** 10.1038/s41598-022-06785-5

**Published:** 2022-02-17

**Authors:** Gun-Soo Park, Jin-Soo Maeng

**Affiliations:** 1 Center for Convergent Research of Emerging Virus Infection, Korea Research Institute of Chemical Technology, Daejeon, 34114 Republic of Korea; 2 Research Division of Food Convergence, Korea Food Research Institute, Wanju-gun, Jeollabuk-do 55365 Republic of Korea

**Keywords:** Biological techniques, DNA, RNA

## Abstract

Isothermal nucleic acid amplification methods have been successfully developed and applied for diagnostic purpose, especially for detection of pathogens. However, amplicon size of such methods is relatively short (< 500 bp) to limit their application for long amplicon production that can be used for various downstream applications including genomic surveillance of pathogens. To fill the gap, we developed a method for specific amplification of kilobases-long target sequence from RNA templates. This method, named CREA, utilizes sequence specific recombination of Cre recombinase to generate circular intermediate template for subsequent RCA reaction. CREA with SARS-CoV-2 *spike* template could amplify ~ 2.9 kb target and up to ~ 1.9 kb amplicon was able to produce in sufficient amount for general cloning. Each step of CREA procedure was thoroughly analyzed to provide directions for further optimizations. Furthermore, we evaluated a variation of CREA which utilized DNA ligase.

## Introduction

Production of target nucleic acid is the starting point of many molecular biology studies. Such nucleic acid can be obtained by extraction from cultured bacteria or mammalian cells, or by in vitro amplification from small amount of nucleic acids. Polymerase chain reaction (PCR) is the representative method for the later. PCR utilizes two primers that bind to each end of target amplicon and a thermophilic DNA polymerase that is active during thermal cycling—oscillation of reaction temperature for template denaturation, primer annealing and polymer extension^1^. PCR is current gold-standard of such technique because of its simplicity, sensitivity, specificity and versatility that can generate several kilobases long products.

Another usual intention of in vitro nucleic acid amplification is to detect target nucleic acid for diagnostic purposes, generally termed as nucleic acid amplification tests (NAAT). Targets of NAAT can be genomic fragments of infectious entities or biological markers of a disease. PCR based techniques are most widely used for NAAT, yet necessity of expensive thermal cyclers and accompanying facilities hinder its use for point-of-care tests (POCT). To overcome this caveat and apply NAAT for POCT, many isothermal nucleic acid amplification methods have been developed^2,3^. Some of representative techniques for isothermal NAAT are loop-mediated isothermal amplification (LAMP)^4^, recombinase polymerase amplification (RPA)^5^, nucleic acid sequences based amplification (NASBA)^6^ and helicase-dependent amplification (HDA)^7^.

While above mentioned isothermal NAAT methods typically target small (< 500 bp) amplicon, some isothermal nucleic acid amplification methods can target much larger templates. One of such methods is rolling circle amplification (RCA). RCA take advantage of strand displacement activity of some DNA polymerases to generate long repeating product from circular DNA template. Typical RCA amplifies circular single stranded DNA (ssDNA) to produce long concatemer. Efficient amplification of repeated sequence by RCA have been utilized not only for detection of target sequence, but also for production of DNA nanoassemblies^8,9^. A variation of RCA, named ramified RCA or hyperbranched RCA (HRCA), uses multiple primers to produce concatemeric dsDNA with various sizes. As a result, HRCA shows improved amplification efficiency^10,11^. A mechanism behind HRCA, the branched amplification through strand displacement, was also adapted for whole genome amplification (WGA) using random primers. This technique, named multiple displacement amplification (MDA), can produce long and non-biased replicates of genomic contents and is routinely used for whole genome sequencing^12–14^. Notably, an improved method of RCA was reported to overcome heat induced pre-annealing step required for double stranded DNA (dsDNA) template and improve dsDNA production using T4 gene 32 protein^15^.

In spite of the assortment of isothermal nucleic acid amplification methods, their application for rather general downstream applications such as cloning is restricted. For NAAT oriented methods, the target amplicon size is limited. Template should be in circular form for RCA and HRCA. Additional steps are required to retrieve specific target from WGA product as it is a mixture of heterologous sequences from random primers. This gap between PCR and isothermal method, amplification of long and specific target sequence from linear template, still remains.

In this study, we developed and evaluated an isothermal method to amplify an arbitrary long (> 1 kb) amplicon from a linear RNA template. Here, we utilized Cre recombinase which exchange dsDNA strands in a directional manner by targeting specific sequence, called *loxP*^16^. This Cre-*loxP* system have been widely used to generate genetic mouse models^17^. We took advantage of Cre recombinase’s site-specific feature to generate specific circular DNA template for RCA reaction. This method, named Circularization-RCA for Extended Amplicon (CREA), would be a useful substitute of PCR in resource-limited settings.

## Results

### Development and optimization

**Figure 1:**
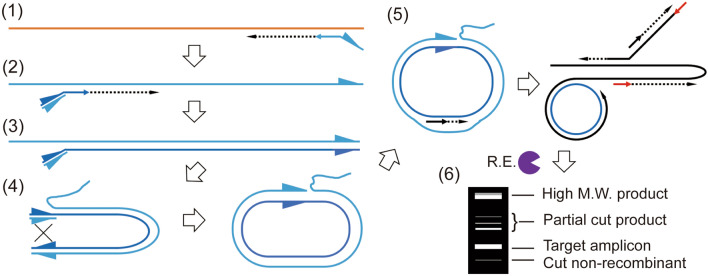
Process of CREA reaction. Numbered steps are described in the main text. *loxP* sequences are designated by right triangles so that the directionality of *loxP* sequences are indicated. In step (5), each RCA primer is indicated by black or red arrow. *R.E.* restriction enzyme.

**Figure 2:**
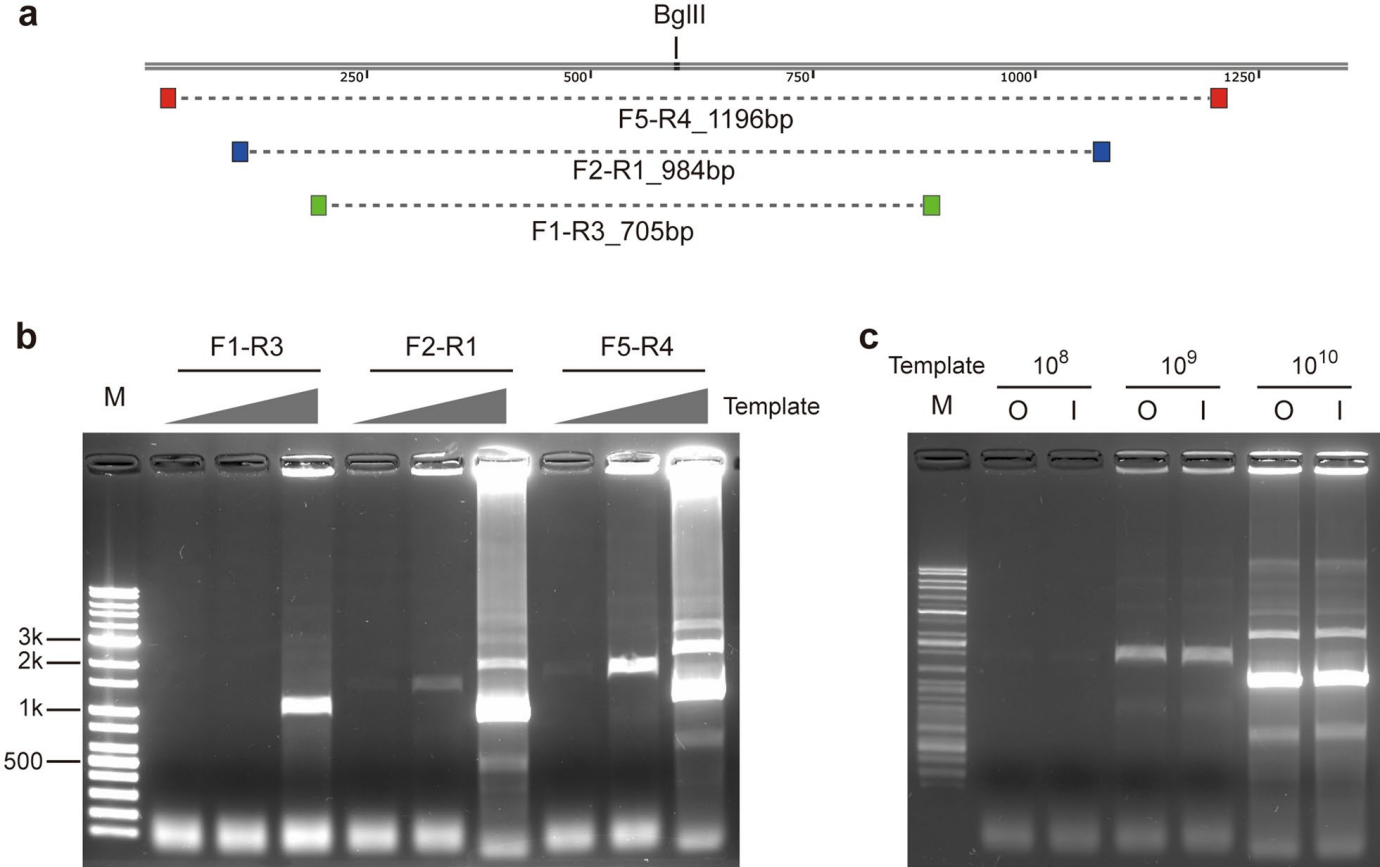
CREA targeting hCoV-OC43 *nucleocapsid*. (**a**) Position of loxP-primers and target length of each primer sets are designated. Position of selected restriction enzyme (BglII) is also marked. (**b**) CREA reaction by primer sets using different template copies of 4.3 × 10^8^, 4.3 × 10^9^ and 4.3 × 10^10^. The same binding region of loxP-primers are chosen for RCA. Sizes of some marker bands are indicated. (**c**) CREA on F5-R4 target with different RCA primer sets by template copies. “O” stands for outer primer set which used F5-R4 RCA primers. “I” stands for inner primer set which used F1-R3 RCA primers. Template copy number set is the same as (**b**). M; marker. Because of non-uniform incorporation of SYBR green I added to the sample before gel loading, disturbance or inaccurate migration occurred.

Process of CREA is shown in Fig. 1; (1) reverse transcription (RT) from reverse primer conjugated with reverse complementary *loxP* sequence (reverse loxP-primer) at 5′ end; (2) RNase H activity of separate enzyme or reverse transcriptase removes bound RNA so that forward primer with 5′ *loxP* sequence pre-annealed with reverse complementary loxP (loxPrc) oligonucleotide (forward loxP-primer) binds to the first strand cDNA; (3) 2nd strand synthesis from forward primer; (4) Cre recombinase make single strand DNA template for RCA reaction; (5) RCA reaction produce long DNA, (6) specific amplicon can be identified by agarose gel electrophoresis after restriction enzyme treatment targeting the amplicon. Typical agarose gel electrophoresis result would show high molecular weight product which are presumably ssDNA, completely or partially cut dsDNA target amplicon, and 2nd strand which are not recombined by Cre. Following assay development and optimization experiments were performed with ~ 10^10^ copies in vitro transcribed (IVT) RNA of hCoV-OC43 *nucleocapsid* and its F5-R4 region (Fig. 2).

Initially, each steps of RT, RNase H treatment, 2nd strand synthesis, recombination and RCA were performed separately (see Supplementary Methods). M-MLV reverse transcriptase (RTase) and phi29 DNA polymerase (DNAP) were chosen as they are widely used for RT and RCA, respectively. Positive results shown after the amounts of each loxP-primers and Cre are adjusted to 0.2 pmol and 2 U respectively for 20 μl reaction volume. At this point, we investigated buffer compatibility. Recombination of Cre was robust in various buffers. As comparing buffers for other enzymes, M-MLV RTase and phi29 DNAP, target amplicon was obtained when phi29 DNA polymerase’s buffer was used as a base (Supplementary Fig. 1). Subsequently, reaction steps and times were optimized to omit RNase H treatment and to combine 2nd strand synthesis and recombination (Supplementary Fig. 2). While separately conducted RCA showed better yield, no significant improvement of sensitivity was observed (Supplementary Fig. 3). Therefore, the RCA reaction was also merged with 2nd strand synthesis-recombination step to make whole CREA process as simple as possible. Finally, the amount of loxP-primers was adjusted to 1 pmol (Supplementary Fig. 4; Supplementary Table 4).

### Sensitivity and size limit of CREA

To evaluate sensitivity and primer dependence of CREA, three primer sets were compared (Fig. 2a). Interestingly, the primer set of the longest amplicon, F5-R4, turned out to be most sensitive among three primer sets while the shortest F1-R3 is least sensitive (Fig. 2b). However, the sensitivity of CREA was not significantly affected by choice of RCA primers (Fig. 2c). As a result, sensitivity and yield of CREA is loxP-primer dependent. Yield and sensitivity of CREA by incubation time of after-RT step were evaluated and the sample with over-night incubation showed the best result in both aspects (Supplementary Fig. 5a).

**Figure 3:**
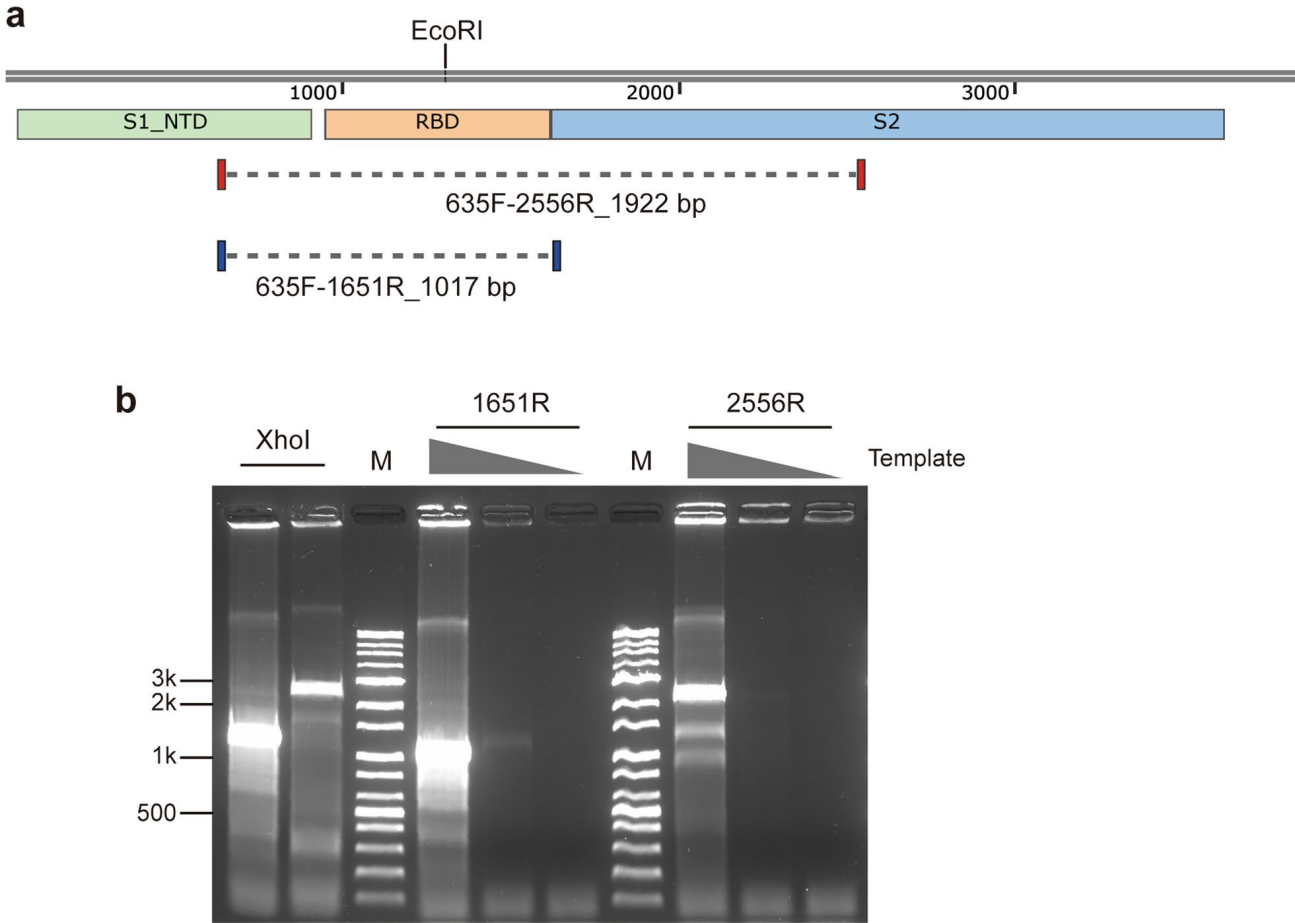
CREA targeting SARS-CoV-2 *spike*. (**a**) Position of loxP-primers and target length of each primer sets are designated. Domains of Spike and position of selected restriction enzyme (EcoRI) is also marked. (**b**) CREA reaction by primer sets using different template copies of 5 × 10^10^, 7 × 10^9^ and 7 × 10^8^. 635F-1651R pair was used for RCA primer. For XhoI cut set (from left, 1651R and 2556R), 5 × 10^10^ copies of template were used. Sizes of some marker bands are indicated. M; marker. Because of non-uniform incorporation of SYBR green I added to the sample before gel loading, inaccurate migration occurred.

To evaluate target size limit of CREA, primer sets targeting *spike* of SARS-CoV-2 were tested. As a result, target size up to ~ 2.9 kb was amplified by CREA (Supplementary Fig. 6). Notably, primer dependent efficiency of CREA was repeatedly shown. To test effect of target size to the sensitivity of CREA, two primer sets with target size ~ 1 kb and ~ 1.9 kb including receptor binding domain of SARS-CoV-2 *spike* were compared (Fig. 3a). Both primer sets showed similar efficiency and sensitivity (Fig. 3b). In addition, proper target amplicon bands were obtained regardless of choice of a restriction enzyme either targeting middle of amplicons (EcoRI) or primer-incorporated sequence (XhoI).

Finally, to confirm the identity of amplified fragments, target amplicon bands were extracted and sequenced after cloning to pBluescript II KS + plasmid. Blast results indicates the bands were indeed from target regions (Supplementary Fig. 7).

### Dissecting CREA steps

As previous results indicate, limit of detection (LoD) of CREA is not so practical for current settings, giving positive results from ~ 10^8^ copies of input template and ~ 10^9^ to ~ 10^10^ copies of input template were required to obtain processible products. To troubleshoot this caveat, efficiency of each steps of CREA was investigated.

First, efficiency and effect of target size on RT was evaluated with 1 pmol of SARS-CoV-2 *spike* 2082R primer and quantitative PCR (qPCR) primer sets targeting various region of the same gene (Supplementary Table 1). 7 × 10^9^ copies of IVT RNA was subjected to RT. M-MLV RTase with buffer-reaction volume conditions of CREA or product’s manual were compared. RT with Superscript IV (SSIV, Invitrogen) sample was added as an improved RT condition. Distance between 5′ residue of qPCR forward primers and reverse transcription primer are designated in the table. Either M-MLV RTase and SSIV showed reduced efficiency for long cDNA production. In addition, overall efficiency of M-MLV RTase was reduced in CREA condition. Simple substitution of M-MLV RTase to other RTase in current CREA setting did not improved the result regardless of RNase H supplementation for which compensate other RTases’ reduced or removed RNase H activity (Supplementary Fig. 8).

Second, efficiency of Cre recombinase was assayed. 1 × 10^4^ copies of PCR product from loxP-primers targeting hCoV-OC43 *nucleocapsid* F5-R4 region was subjected to recombination. The recombination was performed after 30 min recombination in 1 × phi29 DNAP buffer with final 10 mM DTT at 37 °C. The efficiencies of recombination by different conditions were compared by recombinant specific qPCR (Supplementary Table 2). Interestingly, activity of Cre was severely inhibited by glycerol. This inhibition by glycerol, in addition to relatively small amount of Cre recombinase compare to the amount of loxP-primers result in low recombination efficiency (Supplementary Table 3).

Next, overall efficiency for generation of RCA template was evaluated. RT products in CREA condition from 4.3 × 10^8^ copies of hCoV-OC43 *nucleocapsid* IVT RNA are made using 0.2, 1, or 2 pmol of each loxP-Primers. Various primer concentrations were compared as it affects not only RT efficiency but also recombination as Cre-mediated recombination is reversible process that the number of *loxP* target should be matched to the amount of Cre proteins for maximum efficiency. Half (8.75 μl) of RT product was incubated for one hour with 2.5 U of phi29 DNAP and 1 U of Cre without RCA primers in 10 μl final volume (Supplementary Table 4). The copy number of recombinant specific qPCR target was high considering previously evaluated RT and recombination efficiencies, indicating some RCA reaction occurred. Decreased RT efficiency compare to above SARS-CoV-2 *spike* used experiment may from *loxP* conjugation to primers, different template-primer sequences, or error in IVT RNA concentration measurement.

Finally, sensitivity of RCA reaction was evaluated. Buffer condition was matched with CREA including exclusion of BSA as BSA was rather inhibitory to generate dsDNA product (Supplementary Fig. 2b). Serial dilutions of PCR-Cre recombinant of hCoV-OC43 *nucleocapsid* F5-R4 amplicon were subjected to overnight RCA reaction at 37 °C. F1-R3 RCA primers were pre-annealed by 2 min incubation at 95 °C. Strikingly, proper RCA product was shown only for the sample with 4.8 × 10^8^ copies input recombinant template (Supplementary Fig. 9). In addition, 10% glycerol was inhibitory for the phi29 DNAP mediated RCA as no amplicon band is shown. T4 Gene 32 protein addition to 500 ng/μl concentration was also inhibitory, in line with inhibitory effect of T4 Gene 32 protein to CREA process (Supplementary Fig. 3).

As a result, RCA is the bottleneck of CREA’s LoD while RT and recombination also have capacity to be improved. Specifically, inhibitory effect of glycerol to recombination and RCA was observed. With the result that the RCA is the bottleneck process, CREA with phi29 DNAP optimized 4 mM DTT concentration was evaluated. 10 mM final DTT concentration was matched to reverse transcriptase because we initially hypothesized RT step as the limiting step. However, no significant improvement was observed (Supplementary Fig. 5b).

### Ligase mediated CREA

**Figure 4:**
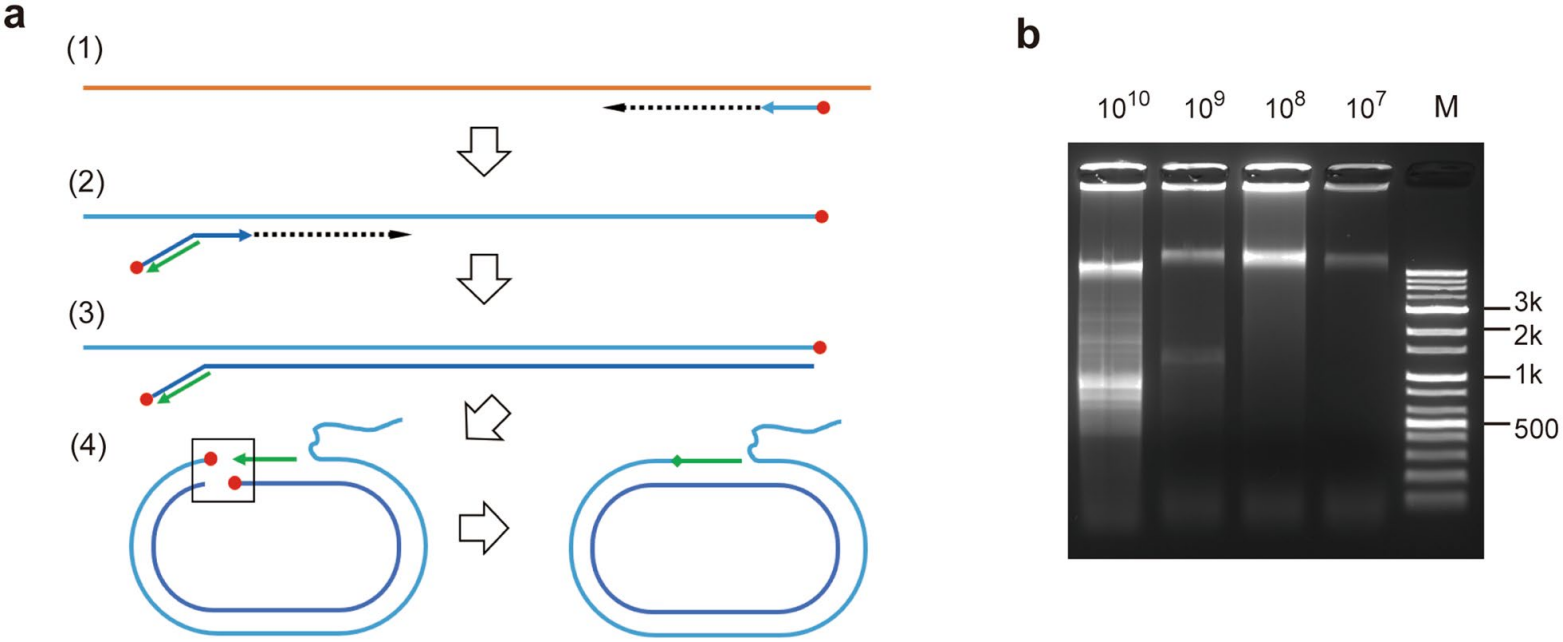
Ligase-CREA. (**a**) Schematic process of ligase-CREA. 5′-phosphate groups are designated with red circle. Ligation target site is marked with a box. (**b**) Sensitivity of ligase-CREA was evaluated with 1/10 serial-diluted templates from 3.1 × 10^10^ to 3.1 × 10^7^ copies. Lower-sized target band of 3.1 × 10^10^ copies template input sample is due to migration disturbance of incorporated SYBR green I since the ligase-CREA product with the same input template showed the same sized band as the product of Cre mediated CREA in the post-stained gel (Supplementary Fig. 10b). *M* marker.

One of intrinsic limitation of CREA mechanism which would negatively affect its sensitivity is reversible nature of Cre mediated recombination. As a method to overcome this caveat, ligation mediated circularization was tested. In this variation of CREA (ligase-CREA), T4 DNA ligase is adapted to irreversibly ligate naturally formed double stranded reverse primer binding region after 2nd strand synthesis and 5′ portion—*loxP* sequence part in this study—of pre-annealed forward primer (Fig. 4a). Here, loxPrc annealed, 5′ phosphorylated forward loxP-primer and 5′ phosphorylated reverse primer without 5′ reverse complementary *loxP* sequence compose circularization primer set. Targeting hCoV-OC43 *nucleocapsid* F5-R4 region, corresponding circularization primers and F1-R3 RCA primer pair were used for following experiments.

LoD of ligase-CREA was not improved so that the target amplicon band was notable from ~ 10^9^ copies of template (Fig. 4b). One notable difference compare to Cre mediated CREA is the rather uniform signal intensity of high molecular weight products shown near wells from as little as ~ 10^7^ copies of template. As a reason, we speculate that Cre may bind to temporal double-stranded *loxP* portion of RCA template to hinder phi29 DNAP activity to reduce high-molecular weight product formation in Cre mediated CREA. Moreover, some non-specific bands or signals are shown. This may from running polymerization or non-specific ligation.

In addition, we tested if nick formation during circularization can improve LoD of ligase-CREA as phi29 DNAP can detect and start polymerization from nicked dsDNA. By using 5′ non-phosphorylated reverse primer, nick is induced between 5′ of reverse primer and 3′ of loxPrc oligo annealed to forward primer. Since this nick is naturally formed in RCA template, loss of efficiency by RCA primer annealing can be avoided. However, nick induction did not improved LoD or yield of ligase-CREA (Supplementary Fig. 10a). One possible reason would be reduced ligation efficiency by lack of one phosphate group.

## Discussion

Production of discrete long amplicon is usually achieved by PCR as it is not readily applicable for previously reported isothermal NAAT techniques. In this study, we developed and evaluated a simple two-step isothermal nucleic acid amplification method that can be performed in relatively low single temperature of 37 °C to produce long dsDNA from arbitrary amplicon on linear RNA template. This method, CREA, utilizes sequence-specific recombination by Cre recombinase to make circular template for following RCA reaction. CREA successfully amplified target amplicon with size up to ~ 2.9 kb and up to ~ 1.9 kb products from hCoV-OC43 *nucleocapsid* or SARS-CoV-2 *spike* gene was successfully sequenced after cloning. In addition, a variation of CREA using T4 DNA ligase was tested. We expect that CREA can substitute PCR for generation of DNA fragments for subsequent molecular biology process such as cloning and sequencing, especially in the places with limited instruments^18^.

In our stream of proof-of-concept experiments, some caveats of CREA methods were found. First, relatively low native M-MLV RTase activity may limit amplicon size. While manufacturers state maximum length of cDNA that M-MLV RTase can generate is longer than 5 kb, we observed dramatic reduction of RT efficiency for targets longer than 1 kb especially in non-optimal condition of CREA buffer (Supplementary Table 1). Using improved RTase may be beneficial, but additional optimization including RNase H treatment method would be required as mere substitution was not successful (Supplementary Fig. 8). Second, LoD of CREA is relatively high compare to other isothermal NAAT methods. We examined each step of RT, recombination and RCA to find the reason. Beside intrinsic limitations such as efficiency RTase and reversibility of Cre mediated recombination, some unexpected factors were found. One of such factors is inhibitory effect of glycerol to Cre and phi29 DNAP. This negative effect of glycerol would be one of reasons of high LoD of CREA as the final concentration of glycerol in our study was 10% because relatively large volume of enzymes, in storage buffers with 50% glycerol, were used. The issue of glycerol concentration would be avoided by using high concentrated enzymes. In addition, native sensitivity of phi29 DNAP mediated RCA was strikingly poor in our hand with LoD of ~ 10^8^ copies of template. Nevertheless, detection-oriented RCA methods using padlock probe are reported to show PCR-comparable LoD with phi29 or other DNAP^10,19–21^, indicating that the LoD of RCA can be improved by proper optimizations. Additionally, dsDNA production by ramification was available with DNAP other than phi29 with higher reaction temperature which would facilitate primer binding. However, direct substitution of phi29 DNAP with other DNAP was not successful for generation of target dsDNA product (Supplementary Fig. 11).

As hinted above, another notable point for improvement is production of dsDNA over ssDNA as many tools for molecular biology such as restriction enzymes are for dsDNA. CREA produce significant amount of high molecular weight product shown near well in agarose gel which is presumably ssDNA. This indicates non-optimal ramification during RCA, presumably due to low primer binding efficiency. While T4 Gene 32 protein is reported to promote primer binding during RCA^15^, it is also reported that the protein facilitates ssDNA production although suggested mechanism is suppression of strand switching^22^. In addition, activity of phi29 DNAP was inhibited by T4 Gene 32 protein (Supplementary Fig. 9). This is also supported by previous report that phi29 DNAP was not applicable for T4 Gene 32 protein mediated amplification for circular DNA^15^. As most part of the intermediate recombinant is hybridized dsDNA of the circular ssDNA and the linear first strand cDNA, active dissociation mechanism may be required for improved LoD and may help dsDNA production. One intuitive way is using thermostable DNAP with two-temperature after-RT step. However, this solution would require fine-tuned optimization such as unit usage of enzymes, buffer composition, usage of T4 Gene 32 protein since direct substitution was not successful as we attempted. In addition, fidelity of DNAP would be important for some downstream applications considering phi29 DNAP has 3′–5′ proofreading exonuclease activity. Otherwise, amplification with other method such as cHDA may be applicable^23^.

In addition, concept of circularization followed by RCA itself may be applied for DNA starting template. In this case, heat induced primer invasion in moderately high temperature around 60–70 °C or pre-annealing process would be required, if an enzymatic method is not applied. In addition, forward loxP-primer would be modified to be a single oligonucleotide that can form stem-loop structure for competitive advantage over self- or hetero-dimer formation. Anyway, procedure including multiple temperature steps would be required as Cre recombinase is not thermostable.

Finally, we would like to emphasize a possible application of CREA—the decentralized genomic surveillance of pathogens. In fact, one of our motivation to develop isothermal method for amplification of properly long target amplicon was current pandemic of COVID-19. Early identification of threatening new variants by genomic surveillance is important for following alarming and update of countermeasures like vaccines^24^. For example, our selection of target amplicon of SARS-CoV-2 *spike* includes its receptor binding domain whose mutation is of special concern because of the possible immune evasion^25^. Such surveillance is usually performed at centralized laboratories since current protocol uses PCR-based amplification of sequencing templates^26,27^. At this point, a good isothermal method for sequencing template preparation would decentralize the place of genomic surveillance to enable earlier alarming of new variants. Two combined methods of an isothermal NAAT and a sequencing technique, LamPORE and LAMP-Seq, were introduced for diagnostic advantages over pooling, multiplexing and specificity^28–30^. MDA is also available for sequencing sample preparation. However, LAMP covers relatively short sequence to call a new variant and additional steps are required during sample preparation or computation for MDA to exclude non-target sequences.

In summary, CREA filled the niche not covered by previously introduced isothermal nucleic acid amplification methods that is to amplify an arbitrary, long, and discrete amplicon from a linear template. We have performed proof-of-concept experiments, demonstrated current limitations and discussed points for improvement. CREA would be a useful tool to generate DNA fragments for basic molecular biology studies such as cloning or for practical use such as monitoring mutations of a pathogen in resource-limited places.

## Methods

### CREA reaction

Before CREA, forward loxP-primer is annealed with loxPrc. For this annealing, forward loxP-primer and loxPrc oligonucleotide are added to final 10 μM in 1 × annealing buffer (10 mM Tris–Cl pH 8, 50 mM NaCl, 1 mM EDTA) than incubated at 95 °C for 5 min in heat block followed by slow cooling to room temperature by turn-off the heat block. Annealed oligo or its 1:10 dilutions is kept in − 20 °C.

Currently optimized CREA procedure is as follows. In RT step, template and each 1 pmol of forward and reverse loxP-Primers were added in RT mix composed of 2 μl of 10 × phi29 DNAP buffer, 1 μl of 10 mM/each dNTP mix, 1.2 μl of 100 mM DTT, 1 μl of M-MLV RTase (Enzynomics, 200 U) and DEPC treated water to final volume of 16.6 μl. RT mix with template and loxP-primers was incubated in 37 °C for 1 h. After RT, each 20 pmol of forward and reverse RCA primers, 1 μl of phi29 DNAP (Enzynomics, 10 U) and 2 μl of Cre recombinase (NEB, 2 U) were added to make 20 μl final reaction volume. This CREA mix was incubated over-night at 37 °C than heat inactivated by 10 min incubation at 70 °C. For ligase-CREA, 1 μl of T4 DNA ligase (Enzynomics) was used instead of Cre recombinase so that the final volume of RT mix was 17.6 μl. 0.2 μl of 100 mM ATP disodium salt was added from RT step for T4 DNA ligase activity. While we used 1 μl of RNA template in TE buffer pH 7.5, using water may be beneficial for larger template volume.

For restriction of CREA product, 1 μl of restriction enzyme was directly added to the reaction tube and incubated 2–3 h in 37 °C. BglII (Enzynomics) was used for hCoV-OC43 *nucleocapsid* templates and XhoI (Enzynomics) or EcoRI (Enzynomics) was used for SARS-CoV-2 *spike* templates.

## Supplementary Information

Supplementary Information.

## Data Availability

No datasets were generated or analyzed during the current study.
