# Supplementary material for: A novel isothermal method for amplification of long specific amplicon from linear template
Source: Sci Rep. 2022 Feb 17;12:2756. doi: 10.1038/s41598-022-06785-5 (PMC8854607; doi:10.1038/s41598-022-06785-5)
Supplement: Supplementary file 1 — Supplementary Information. [file 41598_2022_6785_MOESM1_ESM.pdf]

Supplementary Information for:

A Novel Isothermal Method For Amplification Of Long Specific Amplicon From Linear Template

Gun-Soo Park[1, 2*], Jin-Soo Maeng[1, 2*]

[1] Center for Convergent Research of Emerging Virus Infection, Korea Research Institute of Chemical Technology, Daejeon 34114, Republic of Korea

[2] Research Division of Food Convergence, Korea Food Research Institute, Wanju-gun, Jeollabuk-do 55365, Republic of Korea

* Corresponding authors

Gun-Soo Park (pcdhmk@krict.re.kr), and Jin-Soo Maeng (maengjs@krict.re.kr)

Supplementary Methods

Primer design

CREA related primers are designed as follows; Sequence of *nucleocapsid* of hCoV-OC43 (NC_006213.1) was subjected to Primer3 and forward and reverse primer pairs were picked by target amplicon size. For SARS-CoV-2 (MN908947) *spike*, primers were designed by Primer3 with receptor binding domain as target or manually selected. PCR primers were manually designed. List and sequences of primers used are shown in Supplementary Table 6.

IVT RNA template preparation

cDNA of hCoV-OC43 and SARS-CoV-2 were made from cultured viral RNA using Transcriptor RTase (Roche) following manufacturer's instruction. PCR product of each cDNA and gene with T7 promoter sequence conjugated forward primer was subjected to in vitro transcription using MEGAscript T7 transcription kit (Invitrogen). Full-sized target RNA was extracted using Zymoclean Gel RNA Recovery Kit (Zymo Research) from native agarose gel electrophoresis with 1x MOPS buffer after denaturation with RNA loading buffer of the IVT kit and 10x SYBR green I (Invitrogen). Copy number of extracted IVT RNA was calculated from concentration measured with NanoDrop Lite spectrophotometer.

Step-wise CREA

Step-wise CREA described in this section is as base condition during assay development and optimization. Detailed differences are described in legends of supplementary figures of each experiment. (1) For RT step, template and 0.2 pmol of reverse loxP-Primer were mixed with 2 μl of 10x phi29 DNAP buffer, 1.2 μl of 100 mM DTT, 1 μl of 10 mM/each dNTP mix, 0.2 μl 10 mg/ml BSA, 0.5 μl of M-MLV RTase, and DEPC treated water to final volume of 17.1 μl. RT is done by 1-hour incubation at 37℃. (2) 0.2 μl of RNase H (Enzynomics, 1 U) was added to the sample and incubated for 20 minutes at 37℃. (3) 0.2 pmol of forward loxP-primer and 0.5 μl of phi29 DNAP was added to the sample then the mix was incubated for 1 hour at 37℃ for 2nd strand synthesis. (4) 2 μl of Cre recombinase was added to the sample then the mix was incubated for 1 hour at 37℃ for recombination. (5) To-recombination sample (20 μl) was mixed with each 20 pmol of RCA primers, 0.5 μl of 10x phi29 DNAP buffer, 0.25 μl of 10mM/each dNTP mix, and DEPC treated water to final volume of 22.75 μl. The mixture was incubated at 95℃ for 5 minutes then cooled down in ice for RCA primer binding. (6) The sample was mixed with 0.25 μl of 10 mg/ml BSA, 1 μl of 100 mM DTT, and 1 μl of phi29 DNAP then subjected to over-night incubation followed by 10 minutes heat inactivation at 70℃ for RCA. Restriction was done by directly adding 1 μl of restriction enzyme and

incubating at 37℃.

Agarose gel electrophoresis

0.5x TAE buffer (20 mM Tris-Acetate, 0.5 mM EDTA) was used for agarose gel preparation and running. Corresponding volume of 6x DNA loading buffer was mixed with sample before loading to 1 % agarose gel. For post-stain, agarose gel was incubated in 0.5x TAE buffer with 5000x SYBR green I at room temperature with mild agitation for around 40 minutes. For sample pre-staining, 300x SYBR green I in DMSO was added to the sample with DNA loading buffer to make 10x final concentration. The mix was incubated at least 5 minutes at room temperature before gel loading. Pre-stained gel was cast with 1x RedSafe (iNtRON bio.). 1Kb plus DNA ladder (Biofact) was used as the marker. Gel images were taken by ChemiDoc Touch system (Bio-rad).

Quantitative PCR

qPCR for hCoV-OC43 *nucleocapsid* was performed with Luna Universal qPCR Master Mix (NEB) and 25 μl reaction volume. For other qPCR targets, QuantiSpeed Probe No-Rox kit (PhileKorea) was used with 15 μl reaction volume. Following templates were used as standards of each target; hCoV-OC43 *nucleocapsid* – plasmid with target amplicon (Bioneer), F5-R4 RCA template – nested PCR product from recombination sample, SARS-CoV-2 *spike* – T7-PCR product cloned plasmid. qPCR reaction was performed with LightCycler 96 system (Roche) except for the experiment corresponding to Supplementary Table 2 of which MIC system (BMS) is used.

Reverse transcription efficiency test

For "CREA" sample, the same reaction mix as RT step of optimized CREA procedure was used so that the reaction volume was 16.6 μl. For "MMLV" and "SSIV" samples, IVT RNA was added to final 20 μl of reaction mix as instructed by the manufacturers except the amount of the primer (1 pmol). "CREA" and "MMLV" samples were incubated for 60 minutes at 37℃ and "SSIV" sample was incubated for 10 minutes at 50℃. All samples were directly subjected to RT reaction without primer annealing steps. After RT, enzymes were heat inactivated by 5 minutes incubation at 95℃ then diluted to final 40 μl with water. 2 μl of RT products were used for each qPCR reaction of technical duplicates. Copy number of qPCR template was calculated from average Cq number using slope and Y-intercept value of each standard curve then multiplied by efficiency from each standard as cDNA is ssDNA while standards were dsDNA. Efficiency is the ratio of this copy number and input

volume corresponding IVT RNA amount of 3.5 x 10^8 copies.

RCA efficiency test

To prepare RCA template, gel-extracted PCR product from F5-R4 loxP-primers were subjected to final 40 μl recombination reaction with 2 U of Cre in 1x phi29 DNAP buffer. The recombination reaction was performed by 30 minutes incubation at 37℃ and heat inactivated by 10 minutes incubation at 70℃. The copy number of recombinant DNA was measured by qPCR with recombinant-specific primers and the serial dilutions were made with water.

For RCA reaction, 5 μl of template was mixed with 2 μl of F1-R3 RCA primer mix (10 μM/each) and incubated for 2 minutes at 95℃ for primer pre-annealing. After primer annealing, 2 μl of 10x phi29 DNAP buffer, 1 μl of 10 mM/each dNTP mix, 1.2 μl of 100 mM DTT, 1 μl of phi29 DNAP and water to final 20 μl were mixed. Either of 3 μl of 50% glycerol or 1 μl of T4 gene 32 protein was added to corresponding samples. For undiluted 4.8 x 10^8 copies/reaction sample, 1.5 μl of 10x phi29 DNAP buffer and 1.4 μl of 100 mM DTT were added after primer annealing instead of above-mentioned volume to match buffer composition. RCA was done by over-night incubation at 37℃. Samples were cut by BglII and pre-stained with SYBR green I before gel loading.

Cloning

pBluescript II KS+ plasmid was cut by BamHI, XhoI or EcoRI and subjected to agarose gel electrophoresis to extract cut product. Calf intestine alkaline phosphatase (Takara) was treated as manufacturer's instruction and purified with a gel extraction kit. Gel-extracted CREA product of target amplicon sized band was ligated with plasmid with T4 DNA ligase by 1-hour incubation at room temperature. The ligated samples were transformed to *E. coli* DH5α competent cells. Two positive colonies for each insert was subjected to culture, miniprep and sequencing.

Manufacturers of selected reagents

Some reagents' manufacturers not stated are indicated here. T4 Gene 32 protein (10 mg/ml) – Enzynomics, Klenow fragment (5 U/μl)– Enzynomics, Bsu DNA polymerase, large fragment (5 U/μl) – NEB, BstLF (8 U/μl) – Enzynomics, Bst 2.0 WarmStart (8 U/μl) – NEB, Bst 3.0 (8 U/μl) – NEB, Vent(exo-) DNA polymerase (2 U/μl) – NEB, AMV Rtase (10 U/μl) – NEB, TopScript II (200 U/μl) – Enzynomics, TopScript III (200 U/μl) – Enzynomics.

Supplementary Tables

Supplementary Table 1. Reverse transcription efficiency.

	S1 – 1903 bp		S3 – 1329 bp		S4 – 140 bp	
Condition	Copies/PCR	Efficiency	Copies/PCR	Efficiency	Copies/PCR	Efficiency
CREA	1.80×10^8	51 %	1.12×10^8	32 %	1.09×10^8	310 %
M-MLV	3.01×10^8	86 %	4.53×10^8	129 %	2.65×10^9	757 %
SSIV	1.14×10^9	326 %	1.03×10^9	293 %	2.53×10^9	723 %
- PCR titrated copy numbers were multiplied by 'efficiency' of each primer sets' standard curve (see Supplementary Figure 12) since template is ssDNA first strand and standard curve was made from dsDNA templates.<br>- Odd pattern (lower number for S3 than S1) and value (of >100%) of "Efficiency" would be caused from measurement error of IVT RNA by NanoDrop spectrometer due to low concentration, incomplete IVT product, and different sensitivity of PCR with each primer sets.						

Supplementary Table 2. Cre efficiency vs. glycerol.

Sample^	loxP primers	Cre (Unit)	Glycerol (final %)	Cq_average
No LP	-	0.2	0.5	33.25
No LP-glycerol	-	0.2	10	40.13
LP	0.2 pmol/ea.	2	10	37.01
^ LP – loxP-Primer				

Supplementary Table 3. Cre efficiency and sensitivity over dilutions.

Input copies^	Recombinant copies	% Recombination
1×10^8	8.98×10^5	0.898
1×10^5	6.35×10^2	0.635
1×10^4	4.70×10^1	0.470
1×10^3 *	9.01×10^0	0.901
5×10^2 *	2.59×10^1	5.180
2×10^2 *	2.21×10^1	11.037
^ hCoV-OC43 *nucleocapsid* F5-R4 region loxP-primer PCR product.<br>* 1 of 2 replicate in qPCR showed proper Cq value – increased % would be due to by-chance reaction.		

Supplementary Table 4. Efficiency of RT and RCA template generation.

	To RT^		To Recombination^		Of Recombination*	
Primer	Rep1	Rep2	Rep1	Rep2	Rep1	Rep2
0.2 pmol	3.51 %	3.93 %	1.83 %	1.10 %	52.1 %	27.9 %
1 pmol	4.22 %	2.24 %	1.58 %	0.97 %	37.4 %	43.4 %
2 pmol	1.95 %	2.32 %	0.28 %	0.40 %	14.3 %	17.1 %
^ ratio to the input template copy number<br>* ratio of RCA template over RT product						

Supplementary Table 6. Primers

target	Name	Sequence (5' – 3')
hCoV-OC43 Nucleocapsid	T7-BHI-OCN_F	TAATACGACTCACTATAGGGATCCATGTCTTTTAC TCCTGGTAAGCAA
	RI-OCN_R	GGTGAATTCCAGATGCCGACATAAGGTTCA
	LoxP_OCN_F1	ACCTCTATAACTTCGTATAGCATACATTATACGAA GTTATATTCTTGGTTCTCTGGAA
	LoxP_OCN_F2	TAATGGATAACTTCGTATAGCATACATTATACGAA GTTATAGAAATGTTCAAACCAGG
	LoxP_OCN_F5	AATAATATAACTTCGTATAGCATACATTATACGAAG TTATAGCAATCCAGTAGTAGAG
	LoxPrc _OCN_R1	CATTCAATAACTTCGTATAATGTATGCTATACGAA GTTATACTGTCAAACCTAATTGC
	LoxPrc_OCN_R3	GGGAACATAACTTCGTATAATGTATGCTATACGAA GTTATCACCACCAAAATTCTGAT
	LoxPrc_OCN_R4	GCACGCATAACTTCGTATAATGTATGCTATACGAA GTTATTTCTCCTTGTCCATTCTT
	5Phos_LoxP_OCN_F5	/5'-phosphorylation/ ATAACTTCGTATAGCATACATTATACGAAGTTATA GCAATCCAGTAGTAGAG
	RCA_OCN_F1	ATTCTTGGTTCTCTGGAA
	RCA_OCN_F2	AGAAATGTTCAAACCAGG
	RCA_OCN_F5	AGCAATCCAGTAGTAGAG
	RCA_OCN_R1	ACTGTCAAACCTAATTGC
	RCA_OCN_R3	CACCACCAAAATTCTGAT
	RCA_OCN_R4	TTCTCCTTGTCCATTCTT
	5Phos_OCN_R4	/5'-phosphorylation/ TTCTCCTTGTCCATTCTT
	OCN_PCR_F	AGCAACCAGGCTGATGTCAATACC
	OCN_PCR_R	AGCAGACCTTCCTGAGCCTTCAAT
OCN_F5-R4 RCA template	OCN_54_CrCh_F	TATGAGTCCAAAACCACAGCGT
	OCN_54_CrCh_R	CCACTTGAGGATGCCATTACCA
	OCN_54_CrCh_Pr	/FAM/ CGATTTCCAGAGGACGCTCTACTACTGG /BHQ1/
	OCN_54_CrCh_Nest_F	GCCCCAATAAACAATGCACTGT
	OCN_54_CrCh_Nest_R	CCTGATGGTTGCTGAGAGGTAG
SARS-CoV-2 spike	NotI-T7-S2S_F	AATGCGGCCGCTAATACGACTCACTATAGCTTG GCAAACCACGCGAA
	XhoI-S2S_dnR	GAACTCGAGCGGTATCGTTGCAGTAGC
	LoxP_S2S_24F	TCTTGTATAACTTCGTATAGCATACATTATACGAA GTTATCTCGAGGCCACTAGTCTCTAGTCAGTGT
	LoxP_S2S_635F	CACGCCATAACTTCGTATAGCATACATTATACGAA GTTATCTCGAGTAGTGCGTGATCTCCCTCAG
	LoxP_S2S_1632F	CACGCCATAACTTCGTATAGCATACATTATACGAA GTTATCTCGAGTGGTTTAACAGGCACAGGTG
	LoxPrc_S2S_1651R	CTCAGTATAACTTCGTATAATGTATGCTATACGAA GTTATCACCTGTGCCTGTTAAACCA
	LoxPrc_S2S_2082R	GTGACAATAACTTCGTATAATGTATGCTATACGAA GTTATGGCAATGATGGATTGACTAGCT
	LoxPrc_S2S_2556R	CTTTTGATAACTTCGTATAATGTATGCTATACGAA GTTATTGCACAAATGAGGTCTCTAGCA
	LoxPrc_S2S_3002R	TCTAAGATAACTTCGTATAATGTATGCTATACGAA GTTATAGTCTGCCTGTGATCAACCT
	LoxPrc_S2S_3513R	GGTCAAATAACTTCGTATAATGTATGCTATACGAA GTTATGCCAGAGATGTCACCTAAATCA
	LoxPrc_S2S_ds76R	CGGTATATAACTTCGTATAATGTATGCTATACGAA GTTATGCATCCTTGATTTCACCTTGCT
	RCA_S2S_635F	TAGTGCGTGATCTCCCTCAG

	RCA_S2S_1632F	TGGTTTAACAGGCACAGGTG
	RCA_S2S_1651R	CACCTGTGCCTGTTAAACCA
	RCA_S2S_2082R	GGCAATGATGGATTGACTAGCT
	SARS2_S1_F	CAATGTTACTTGGTTCCATGCTA
	SARS2_S1_R	GTGGAAGCAAAATAAACACCATC
	SARS2_S1_Pr	/FAM/ ACATGTCTCTGGGACCAATGGTAC /BHQ1/
	SARS2_S3_F	GGTGATTCTTCTTCAGGTTGGA
	SARS2_S3_R	GTACACTTTGTTTCTGAGAGAGG
	SARS2_S3_Pr	/FAM/ CTGGTGCTGCAGCTTATTATGTGGG /BHQ1/
	SARS2_S4_F	GCTGTTTAATAGGGGCTGAAC
	SARS2_S4_R	AATGATGGATTGACTAGCTAC
	SARS2_S4_Pr	/FAM/ TCAGACTAATTCTCCTCGGCG /BHQ1/
loxP	loxPrc	ATAACTTCGTATAATGTATGCTATACGAAGTTAT

Supplementary Figure 1

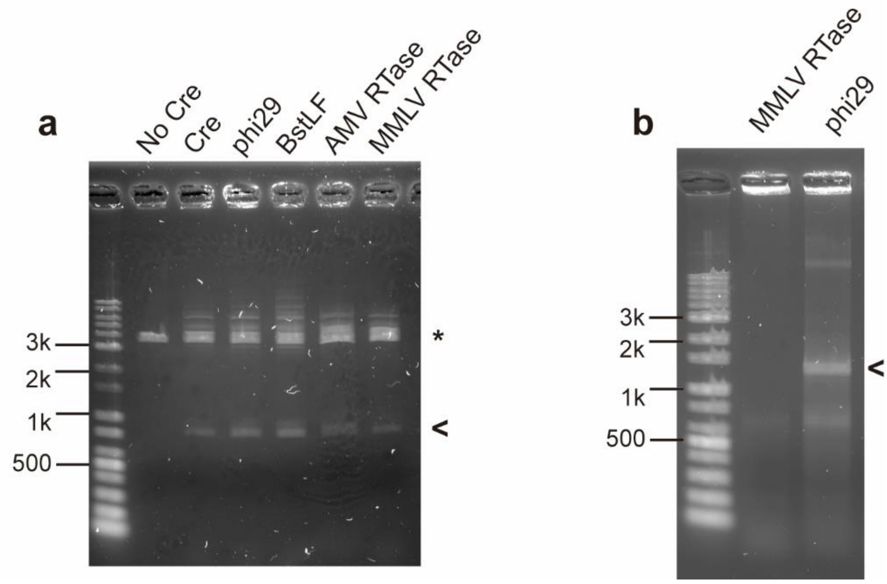

Supplementary Figure 1. Buffer selection for CREA. (a) 100 ng of linearized pLox2+ DNA was incubated with 0.4 U of Cre in 10 μl reaction volume for 60 minutes in 37℃, then heat inactivated at 70℃ for 10 minutes. Agarose gel was post-stained with SYBR green I. Each lane's buffer is as follow; No Cre – in Cre buffer without Cre protein, Cre – in Cre buffer, phi29 – in phi29 DNAP buffer with BSA supplementation to 100 ng/μl, BstLF – in Bst LF buffer with $MgSO_4$ supplementation to 8 mM final concentration of Mg^{2+} ion, AMV RTase – in AMV RTase buffer, MMLV RTase – in M-MLV RTase buffer. Recombination produced 838 bp DNA fragments are designated with "<". Asterisk indicates original pLox2+ size. (b) Whole CREA process was done with indicated enzyme's buffer as base and by step-wise procedure (see Supplementary Methods). 100 ng/μl BSA for both condition and final 10 mM DTT for "phi29" were supplemented during RT to recombination. For RCA, final 8.8 mM Mg^{2+} ion concentration was set for "MMLV" sample with $MgSO_4$. BSA and DTT are supplemented with 25 μl reaction volume corresponding amount for final 100 ng/μl and 4 mM concentration, respectively, in consideration of heat denaturation during RCA primer annealing. Samples loaded for agarose gel electrophoresis after restriction with BglII and the gel is subjected to SYBR green I post-stain. "<" indicates target amplicon.

Supplementary Figure 2

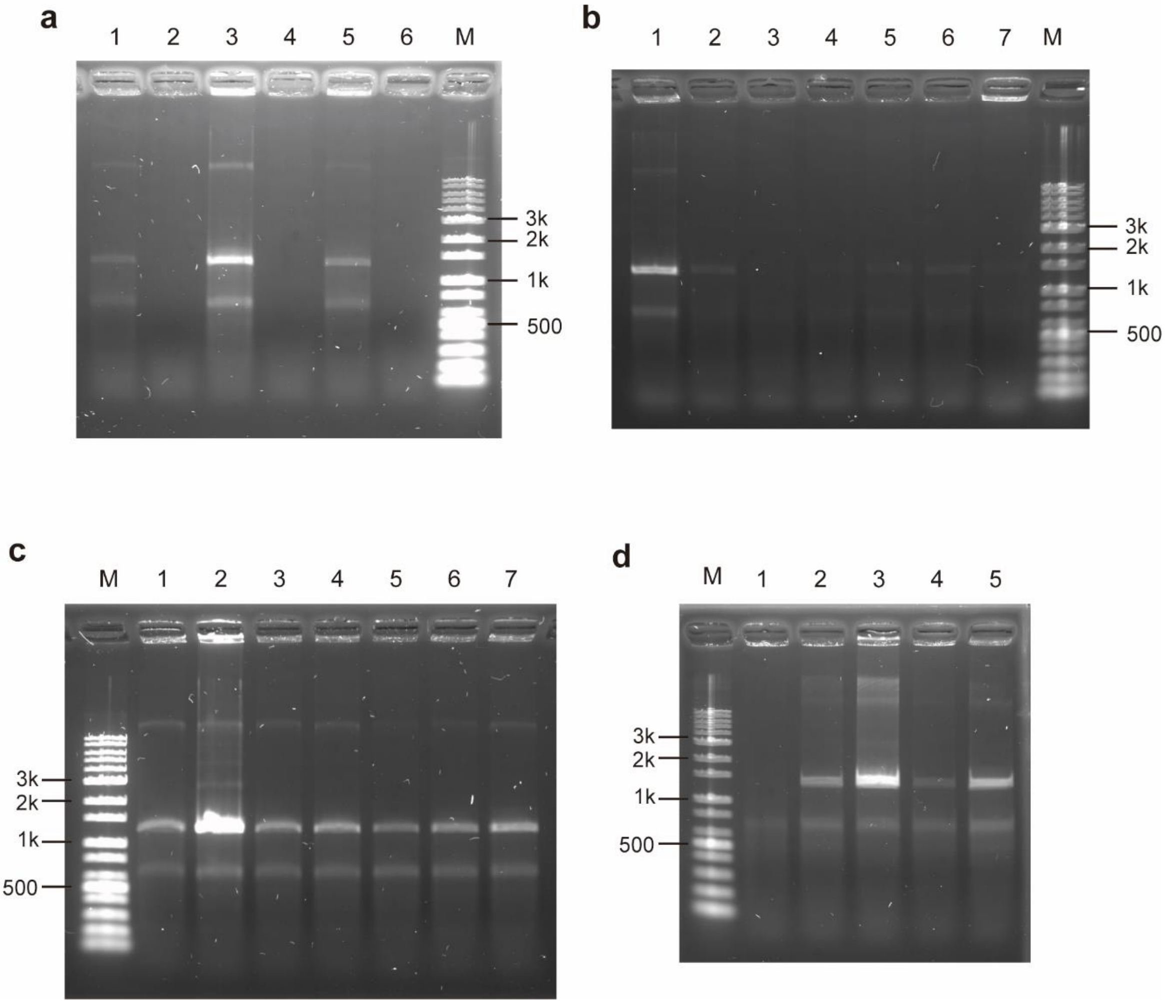

Supplementary Figure 2. CREA steps optimization. SYBR green I post-stained agarose gel images for CREA optimization experiments. For the experiments in this figure, BSA was removed from the reaction buffer condition. RCA was done separately. Explanation of numbered lanes and brief conclusions are explained as follow;

(a) Lane 1 – starting, step-wise reaction control. Lane 2 – RT and RNase H steps are merged. Lane 3 – RNase H treat step is omitted. Lane 4 – RT, RNase H and 2nd strand synthesis steps are merged. Lane 5 – 2nd strand synthesis and recombination steps are merged. Lane 6 – RT, RNase H, 2nd strand synthesis and recombination steps are merged. Total incubation time to recombination was the same for all the samples. RNase H treatment step was removed by this experiment as the tested excessive amount was inhibitory.

(b) Lane 1 – step-wise reaction control. Lane 2 – RT and 2nd strand synthesis steps are merged. Lane 3 to 6 – RT, 2nd strand synthesis and recombination steps are merged with total incubation time of

30 minute, 1, 2, or 3 hours. Lane 7 – Same as lane 6, but with 100 ng/μl BSA supplementation. Here, it is found that RT is inhibited by merging with 2nd strand synthesis. In addition, BSA seems to facilitate RCA itself but somehow reduced dsDNA product formation.

(c) lane 1 – step-wise reaction control. Lane 2 – step-wise reaction but forward loxP-primer was added from RT step. Lane 3 – recombination step time is reduced to 30 minutes. Lane 4 – both 2nd strand synthesis and recombination steps' time are reduced to 30 minutes. Lane 5 to 7 – 2nd strand synthesis and recombination steps are merged with incubation time of 30 minutes, 1 hour or 2 hours. Here, it is found that 2nd strand synthesis and recombination steps can be merged. Forward loxP-primer is added from RT step after this experiment.

(d) lane 1 to 3 – different RT steps incubation time of 10 minutes, 30 minutes or 1 hour with separated 2nd strand synthesis and recombination with 30 minutes of incubation time for each. Lane 4 – step-wise with following incubation times; 30 minutes RT, 10 minutes 2nd strand synthesis, and 30 minutes recombination. Lane 5 – 30 minutes RT step followed by merged 1hr 2nd strand synthesis-recombination step. RCA was done for 4 hours instead of over-night incubation.

As a result, following step-incubation time setting is used for subsequent experiment; 1hr RT and 1hr merged 2nd strand synthesis and recombination. In addition, RTase amount is increased from 100 U to 200 U.

Supplementary Figure 3

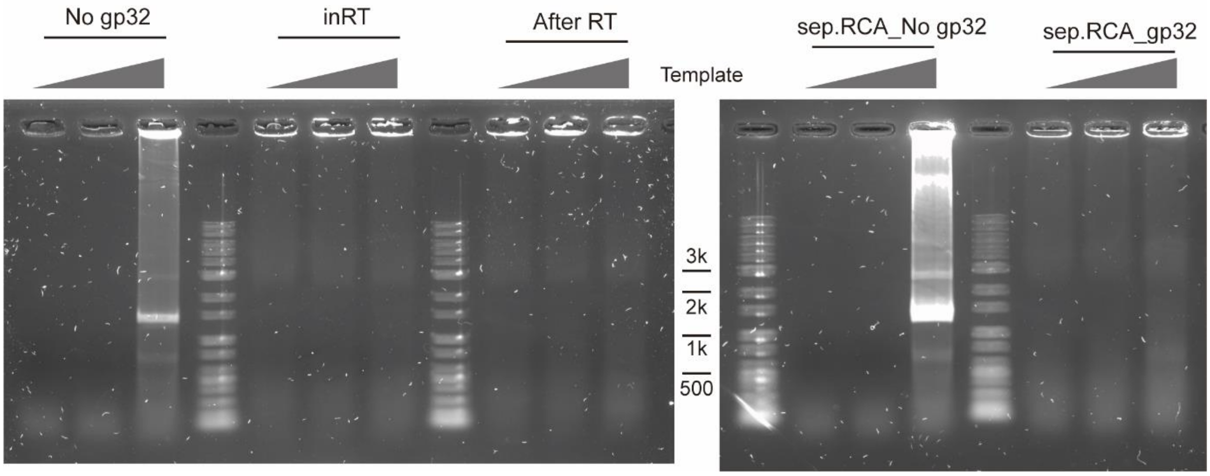

Supplementary Figure 3. CREA steps optimization for merged or separated RCA and by T4 Gene 32 protein (gp32) addition (500 ng/μl). For merged RCA, phi29 DNAP amount added after RT is increased to 10 U and RCA primers are added for the merged step of 2nd strand synthesis to RCA which was done by over-night incubation at 37℃. Input template copy numbers are ~10^8, ~10^9 or ~10^{10} for each set of following conditions; No gp32 – no gp32 is added, inRT – gp32 is added from RT step, After RT – gp32 is added to merged 2nd strand synthesis to RCA step. For separated RCA sets, gp32 was added as 20 μl reaction volume corresponding amount for 25 μl final volume so that its concentration is 400 ng/μl. Here, gp32 blocked production of target dsDNA. RCA step is merged after this experiment for simplicity of whole process as no significant improvement of sensitivity is observed. The agarose gel was post-stained with SYBR green I.

Supplementary Figure 4

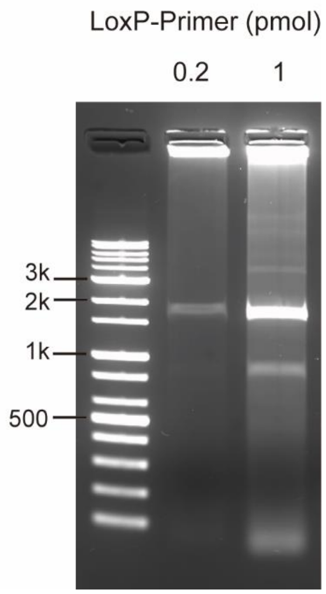

Supplementary Figure 4. CREA with indicated amount of loxP-primers were compared. Here, steps from 2nd strand synthesis to RCA (after-RT) were merged and samples were pre-stained with SYBR green I before gel loading. Inaccurate band size compare to the DNA marker would from non-uniform incorporation of SYBR green I dye.

Supplementary Figure 5

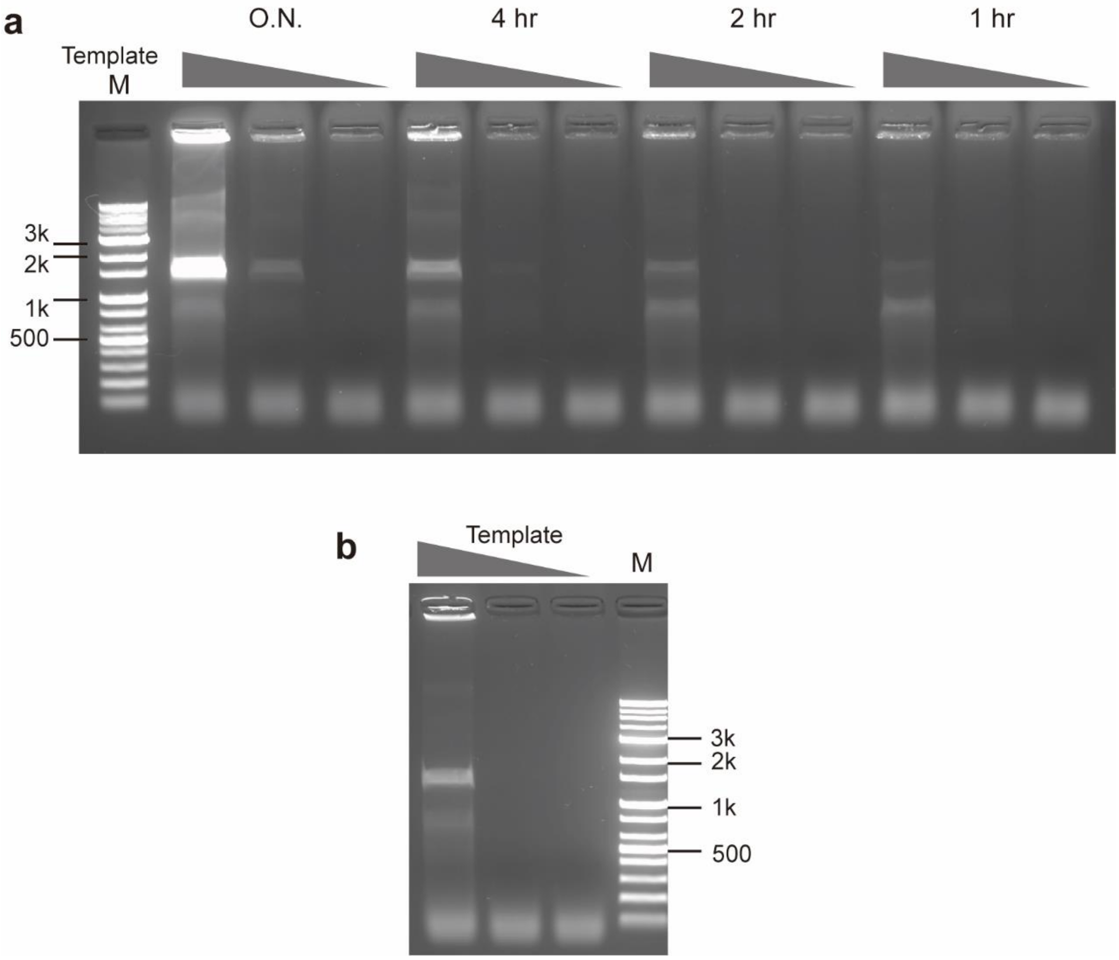

Supplementary Figure 5. (a) CREA results by incubation time of after-RT step. 3.1 x 10^{10} to 10^{8} copies of hCoV-OC43 *nucleocapsid* IVT RNA were subjected to CREA reaction as described in the Methods section of the main text with designated incubation time for after-RT step. F5-R4 pair was used for loxP-primers and F1-R3 primer pair was used for RCA. Target amplicon production was increased by longer incubation time while the intensity of cut non-recombinant bands were rather uniform over the tested incubation times. (b) CREA performed without DTT supplementation so that the working concentration was 4 mM. 3.1 x 10^{10} to 10^{8} copies of hCoV-OC43 *nucleocapsid* IVT RNA were used as templates. No significant improvement was observed compare to CREA with DTT supplementation.

Supplementary Figure 6

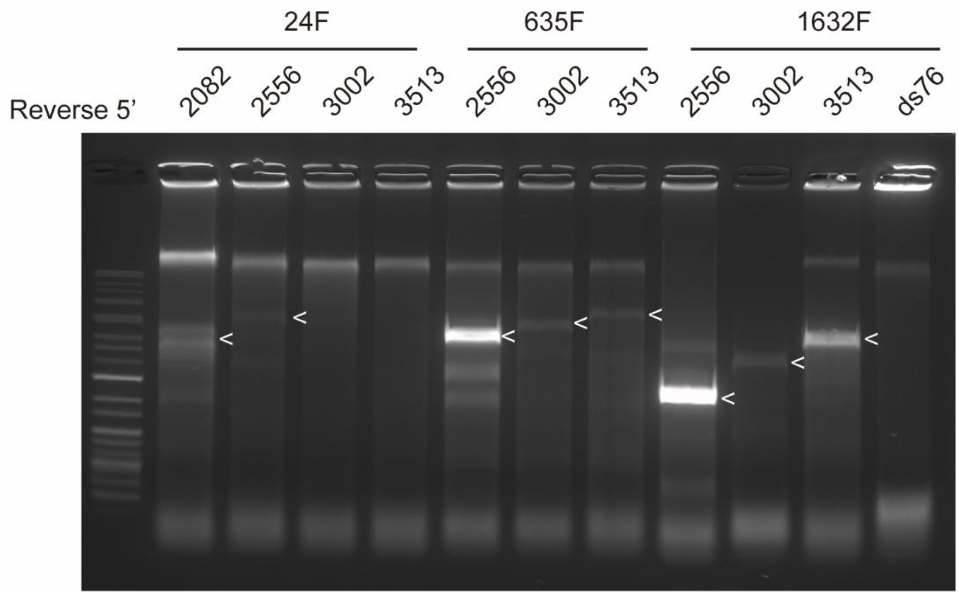

Supplementary Figure 6. CREA with ~10^{10} copies of SARS-CoV-2 spike IVT RNA with indicated forward and reverse loxP-primers. For 1632F loxP-primer used samples, 1632F-2082R RCA primer pair used and BglII was chosen for the restriction except for ds76R loxP-primer sample (BamHI). For other samples, 635F-1651R RCA primer pair and EcoRI were used. Samples were pre-stained with SYBR green I before gel loading and disturbed marker migration occurred due to non-uniform incorporation of SYBR green I dye. The bands of target amplicons are designated with white “<”.

Supplementary Figure 7

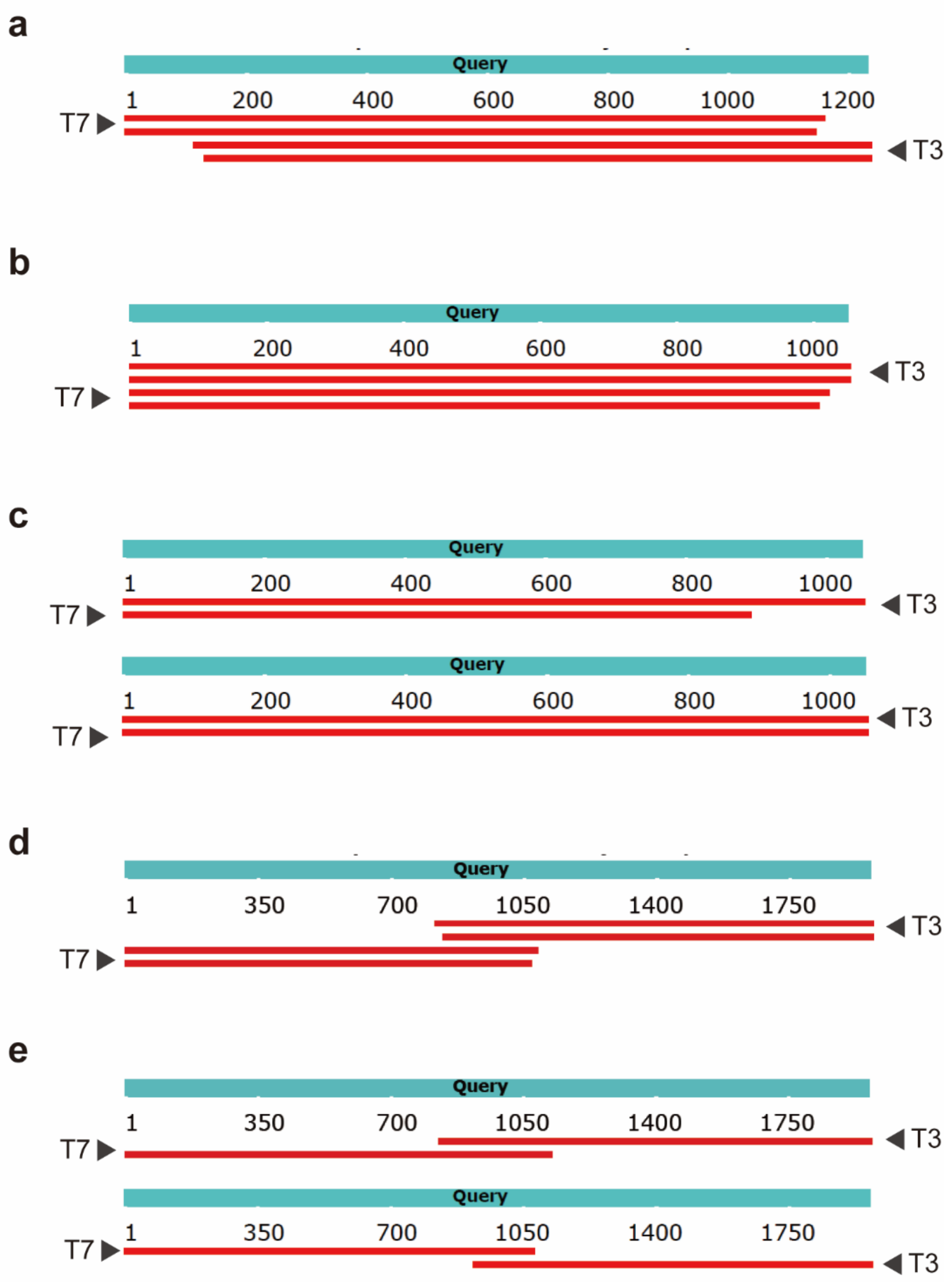

Supplementary Figure 7. BLAST result of cloned CREA products. Two clones were subjected for sequencing for each sample. Primers used for sequencing and their directionality is designated. Target, loxP-primer set and restriction enzymes are as follow; (a) hCoV-OC43 *nucleocapsid*, F5-R4, BglII. (b) SARS-CoV-2 *spike* (S2S), 635F-1651R, XhoI. (c) S2S, 635F-1651R, EcoRI. (d) S2S, 635F-2556R, XhoI. (e) S2S, 635F-2556R, EcoRI.

Supplementary Figure 8

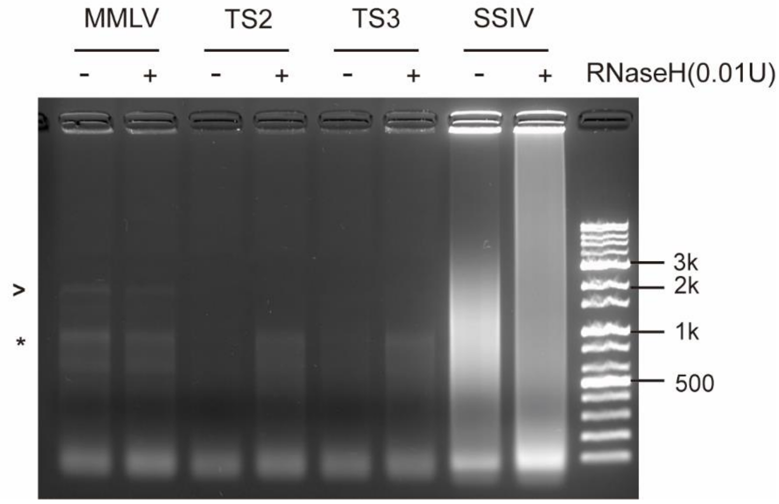

Supplementary Figure 8. RTase compatibility test for CREA with phi29 DNAP and its buffer. 2.7 x 10^{10} copies of hCoV-OC43 *nucleocapsid* IVT RNA, F5-R4 loxP-primer set and F1-R3 RCA primer set are used with 200 U of indicated RTases; MMLV – M-MLV RTase, TS2 – TopScript II, TS3 – TopScript III, and SSIV – SuperScript IV. For RNase H added samples, 0.01U of RNase H was added in after-RT step which is performed for 4 hours at 37℃. Samples were pre-stained with SYBR green I before gel loading. Target amplicon sized band position is designated as ”>” and asterisk (*) designates restriction product of non-recombinant 2nd strand band’s position.

Supplementary Figure 9

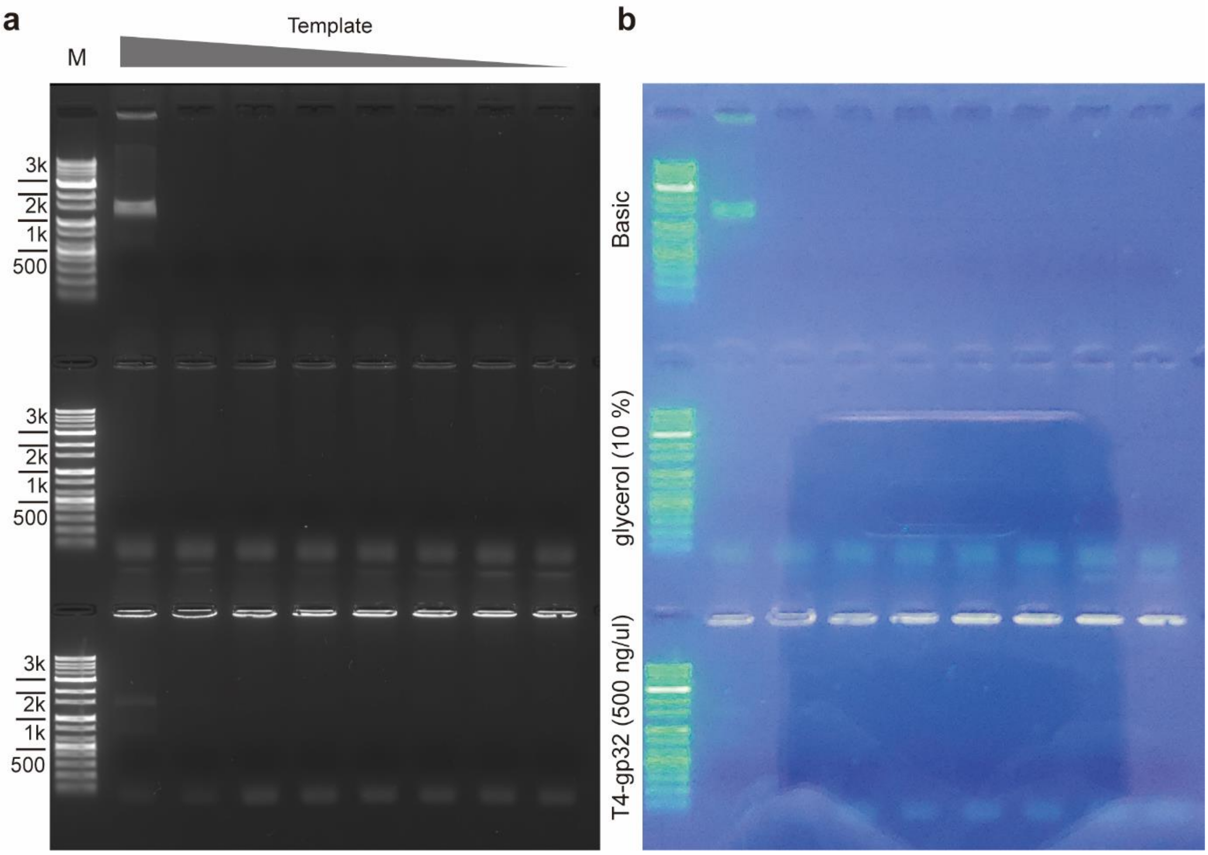

Supplementary Figure 9. RCA sensitivity test. PCR-recombination dsDNA RCA template of hCoV-OC43 *nucleocapsid* F5-R4 region was generated and titrated by recombinant specific qPCR. 10-fold Serial dilutions are made and 4.8 x 10^8 to 4.8 x 10^1 copies templates were used per reaction. While signals shown near well of gp32 added samples in (a) ChemiDoc system image, different color of the signal in (b) cell phone image indicates that the signal would not be from DNA products.

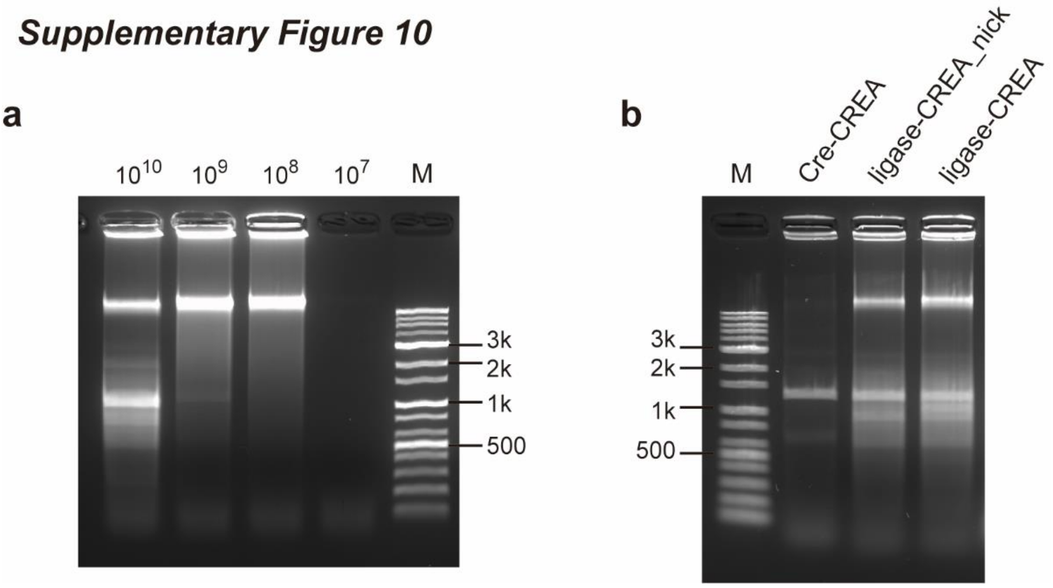

Supplementary Figure 10. Ligase-CREA related experiments. Reagent composition of ligase-CREA is the same as Cre mediated CREA except use of 400 U of T4 DNA ligase instead of 2 U of Cre and addition of final 1 mM of ATP. hCoV-OC43 *nucleocapsid* IVT RNA, F5-R4 loxP-primer set and F1-R3 RCA primer set are used. (a) Ligase-CREA with reverse circularization primer without 5′ phosphorylation over various input template copies of 3.1 x 10^{10} to 3.1 x 10^{7}. Samples were pre-stained with SYBR green I before agarose gel electrophoresis. (b) 3.1 x 10^{10} copies of templates were subjected to designated variations of CREA. The gel was post-stained with SYBR green I to compare migration pattern accurately. M; marker.

Supplementary Figure 11

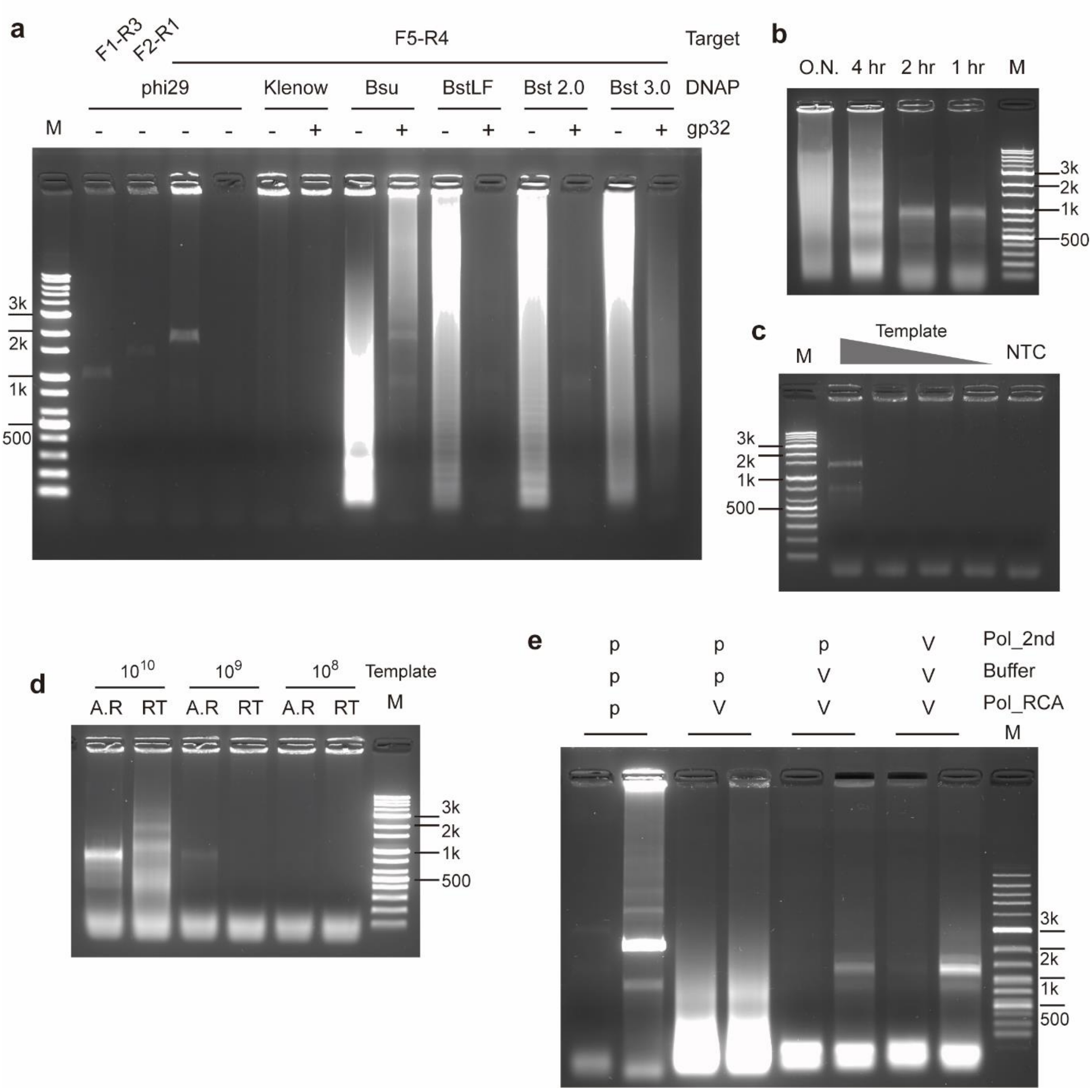

Supplementary Figure 11. Compatibility of different DNA polymerases for CREA.

(a) ~10^{10} copies of hCoV-OC43 *nucleocapsid* IVT RNA was used as a template. Based on step-wise method, 2nd strand synthesis and recombination steps are merged and RCA was done separately using primers targeting the same region of loxP-primers pre-annealed by 5 minutes incubation at 95℃. With designated polymerases' buffer as base, DTT is supplemented to final concentration of 10 mM before RCA and to the concentration in each polymerases' manual after heat induced primer annealing in RCA. For Bst derived polymerases, $MgSO_4$ was added to make 8 mM Mg^{2+} ion concentration and the concentration of dNTP was 1 mM. 1 µl of designated polymerases were used for RCA with while 2nd strand synthesis was done with 0.5 µl of phi29 DNAP. For RCA, 4 hours incubation at 37℃ or 48℃ for Bst derived polymerases as they are thermostable and Bst 2.0 was

WarmStart version which is active above 45℃. For other polymerases than phi29 DNAP, RCA was done with half of volume for each of with or without gp32 (500 ng/μl) added after primer annealing. Sample amount is matched for agarose gel electrophoresis. AMV RTase was used instead of M-MLV RTase for the 4th lane of phi29 DNAP used samples from left.

(b) The incubation time of after-RT step of CREA reactions with Bsu DNA polymerase was evaluated. Each reaction was performed with 3.1×10^{10} copies of hCoV-OC43 *nucleocapsid* IVT RNA according to optimized CREA described in the Methods section of the main text. F5-R4 pair was used for loxP-primers and F1-R3 primer pair was used for RCA. The buffer accompanied by the Bsu polymerase was used without DTT supplementation and gp32 (500 ng/μl) was added in after-RT step. Specific band is notable up to 2 hours incubation.

(c) RCA efficiency of Bsu polymerase. The experiment was done as described in supplementary methods with following modifications. Initial template amount was 8 μl, the working concentration of DTT was 1 mM, 500 ng/μl of gp32 was added, 1 μl of Bsu polymerse was used and the incubation time was one hour. The template copy number of samples were 4.3×10^8, 4.3×10^6, 4.3×10^4, and 4.3×10^1. NTC; non-template control.

(d) Sensitivity of CREA with Bsu DNAP was evaluated. CREA reactions were performed as (b) with 3.1×10^{10} to 10^8 copies of hCoV-OC43 *nucleocapsid* IVT RNA. gp32 (final 500 ng/μl) was added in after-RT step (A.R) or from the RT step (RT) to test effect of gp32 to reverse transcription. F5-R4 pair was used for loxP-primers and F1-R3 primer pair was used for RCA. After-RT step was done for 2 hours. Overall, sensitivity was not improved compare to CREA with phi29 DNAP.

(e) Vent (exo-) DNAP was evaluated for CREA. ~10^9 copies (left of each set) or ~10^{10} copies (right of each set) of hCoV-OC43 *nucleocapsid* IVT RNA was used as a template and target region was of F5-R4. Based on step-wise method, 2nd strand synthesis and recombination steps are merged and 1 pmol of loxP-primers are used. For RCA, 25 pmol of RCA primers targeting the same region of loxP-primers were pre-annealed by 2 minutes incubation at 95℃ with to-recombination product and primers only then other components are added. 1 μl of 100 mM DTT was supplemented for the sample of which RCA polymerase was phi29 DNAP. The amount of polymerase was 0.5 μl for merged 2nd strand synthesis and recombination step and 1 μl for RCA step. Choices of polymerase for 2nd strand synthesis and RCA and base buffer are designated; p – phi29, V – Vent (exo-). Incubation temperature and time conditions of RCA are 4 hours at 37℃ for phi29 DNAP and 2 hours at 65℃ for Vent (exo-).

M; marker. Different size of the band compare to the DNA marker would be caused by non-uniform incorporation of SYBR green I dye similar to ligase-CREA results (see Supplementary Fig. 10).

Supplementary Figure 12

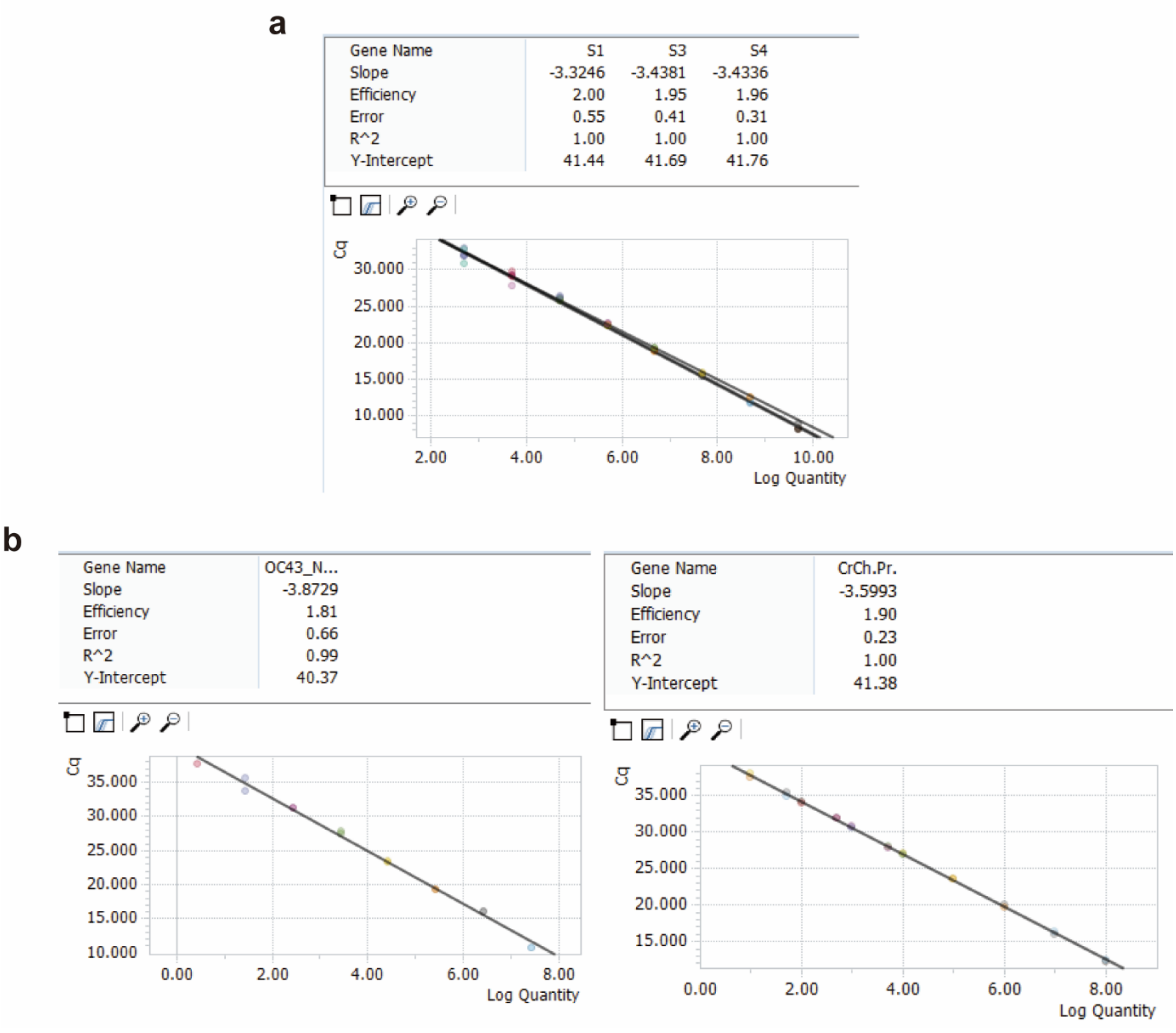

Supplementary Figure 12. Standard curves for titration with qPCR. (a) for S1, S3, and S4 of SARS-CoV-2 spike. (b) for hCoV-OC43 nucleocapsid (left) and its F5-R4 region's recombinant-specific qPCR (right)
